# Using Machine Learning to Predict-Then-Optimize Elective Orthopedic Surgery Scheduling to Improve Operating Room Utilization: Retrospective Study

**DOI:** 10.2196/70857

**Published:** 2025-09-10

**Authors:** Johnathan R Lex, Aazad Abbas, Jacob Mosseri, Jay Singh Toor, Michael Simone, Bheeshma Ravi, Cari Whyne, Elias B Khalil

**Affiliations:** 1 Orthopaedic Biomechanics Lab, Sunnybrook Research Institute, 2075 Bayview Avenue, Suite S620, Toronto, ON, M4N 3M5, Canada; 2 Institute of Biomedical Engineering, University of Toronto, Toronto, ON, Canada; 3 Department of Surgery, Division of Orthopedic Surgery, University of Toronto, 149 College St, Toronto, ON, M5T 1P5, Canada, 1 (416) 946-7957; 4 Department of Mechanical and Industrial Engineering, Faculty of Engineering, University of Toronto, Toronto, ON, Canada; 5 Winnipeg Spine Program, Health Sciences Centre, Department of Surgery, University of Manitoba, Manitoba, Canada; 6 Orthopaedic Surgery Section, Department of Surgery, University of Manitoba, Manitoba, Canada; 7 Division of Orthopaedic Surgery, Department of Surgery, Sunnybrook Health Science Centre, Toronto, ON, Canada

**Keywords:** machine learning, orthopedic surgery, optimization, elective surgery, scheduling, hip and knee arthroplasty

## Abstract

**Background:**

Total knee and hip arthroplasty (TKA and THA) are among the most performed elective procedures. Rising demand and the resource-intensive nature of these procedures have contributed to longer wait times despite significant health care investment. Current scheduling methods often rely on average surgical durations, overlooking patient-specific variability.

**Objective:**

To determine the potential for improving elective surgery scheduling for TKA and THA, respectively, by using a 2-stage approach that incorporates machine learning (ML) prediction of the duration of surgery (DOS) with scheduling optimization.

**Methods:**

In total, 2 ML models (one each for TKA and THA) were trained to predict DOS using patient factors based on 302,490 and 196,942 patients, respectively, from a large international database. In total, 3 optimization formulations based on varying surgeon flexibility were compared: Any (surgeons could operate in any operating room at any time), Split (limitation of 2 surgeons per operating room per day), and multiple subset sum problem (MSSP; limit of 1 surgeon per operating room per day). Two years of daily scheduling simulations were performed for each optimization problem using ML prediction or mean DOS over a range of schedule parameters. Constraints and resources were based on a high-volume arthroplasty hospital in Canada.

**Results:**

The TKA and THA prediction models achieved test accuracy (with a 30 min buffer) of 78.1% (mean squared error 0.898) and 75.4% (mean squared error 0.916), respectively. Any scheduling formulation performed significantly worse than the Split and MSSP formulations with respect to overtime and underutilization (*P*<.001). The latter 2 problems performed similarly (*P*>.05) over most schedule parameters. The ML prediction schedules outperformed those generated using a mean DOS for most scheduling parameters, with overtime reduced on average by 300-500 minutes per week (12‐20 min per operating room per day; *P*<.001). However, there was more operating room underutilization with the ML prediction schedules, with it ranging from 70‐192 minutes more underutilization (*P*<.001). Using a 15-minute schedule granularity with a waitlist pool of a minimum of 1 month generated the ML schedule that outperformed the mean schedule 97.1% of times.

**Conclusions:**

Assuming a full waiting list, optimizing an individual surgeon’s elective operating room time using an ML-assisted predict-then-optimize scheduling system improves overall operating room efficiency, significantly decreasing overtime. This has significant potential implications for health care systems struggling with pressures of rising costs and growing operative waitlists.

## Introduction

Total knee and hip arthroplasty (TKA and THA, respectively) are the gold-standard treatment for end-stage arthritis of the hip and knee joints. These procedures are the first and second most frequently performed in the United States, excluding maternal and neonatal procedures [1]. In 2018, 1.3 million of these procedures were performed in the United States, a 21.9% increase from 2008 [1]. This number will continue to increase globally due to an aging population and the ongoing obesity pandemic [2,3]. The ubiquity of these procedures correlates strongly with their burden on health care systems globally. In the United States, approximately 5% of the gross domestic product is to care for musculoskeletal conditions [4,5]. Despite this spending, wait times for elective surgical procedures in OECD countries continue to increase, conferring extended periods of time with poor quality of life for TKA and THA patients [6,7]. For these reasons, there is a growing interest and research into improving the efficiency and cost-effectiveness of arthritis care [8,9].

Most research has focused purely on the prediction of duration of surgery (DOS) or the optimization of operating room scheduling, ignoring their inherent interrelation [10-14]. More recently, these approaches have been combined to plan post-surgical beds and plan emergency surgeries based on predicted priority with varying results [15,16]. DOS prediction models typically represent a large volume of varying procedures, spanning multiple surgical specialties with limited practical applications. Classic regression modeling in orthopedics has identified age, BMI, surgical procedure, primary or revision, and gender as important variables affecting DOS predictions [17,18]. Machine learning (ML) models have been applied to predict DOS for various surgeries [10,12,19-24]. However, in practice, mean time or surgeon-specific rolling mean time is typically used to generate schedules at the operational level.

Research evaluating the optimization of surgery scheduling has been performed using an average or a randomly sampled (typically from a lognormal distribution) DOS value prior to optimizing a schedule through integer linear programming based on either the multiple knapsack or job-shop scheduling problem [25-27]. Stochastic programming and distributionally robust optimization have also been attempted to mitigate the effects of an uncertain DOS, but these approaches assume a distribution of DOS rather than using specific features to aid in prediction [28].

To our knowledge, no prior work has combined patient-level DOS predictions with schedule optimization to create an optimized surgical schedule at the operational level. It is known that neural networks are strong predictors of DOS; however, their realizable improvements when implemented over various surgical schedule optimization problems, while performing simulations accounting for real-world constraints, remain unclear [23,24]. The primary aim of this paper was to determine if a 2-stage approach using an ML model for prediction of DOS paired with schedule optimization improves operating room overutilization or underutilization compared to using the mean DOS. Secondary objectives were to determine the effect of schedule granularity and length of surgeon waitlist on scheduling accuracy.

## Methods

### Ethical Considerations

This retrospective study analyzed deidentified American College of Surgeons National Surgical Quality Improvement (ACS NSQIP) data. The study was approved by the Institutional Research Ethics Board (Sunnybrook Health Sciences Center, Project REB ID #4899). Informed consent was waived due to the secondary, minimal-risk nature of the analysis. No local institutional patient data were used. Privacy and confidentiality were protected in accordance with institutional and journal policies. Data use complied with NSQIP data-use agreements. The study adhered to the Declaration of Helsinki.

### Setting

**Figure 1.**
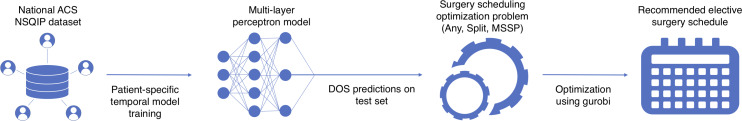
High-level overview of the predict-then-optimize approach. ACS NSQIP: American College of Surgeons National Surgical Quality Improvement Program; DOS: duration of surgery; MSSP: multiple subset sum problem.

Population-level data from the ACS NSQIP database were used to generate prediction models. This database compiles patient and outcome data following surgery from over 700 hospitals in North America, capturing over 1 million surgeries per year, with a high level of accuracy [29]. The database was queried for all TKA and THA surgical procedures performed between 2014 and 2019. The actual DOS times as reported in the ACS NSQIP dataset were used to inform simulated daily operating room schedules. A flowchart that helps visualize the overall 2-stage approach is shown in Figure 1.

### Duration of Surgery Prediction

Data pertaining to 302,490 TKA and 196,942 THA procedures performed during this period as identified from the ACS NSQIP database were used to train models to predict DOS. DOS predictions were done as per Abbas et al [23,24]. They identified a PyTorch multilayer perceptron model that outperformed 10 alternative ML models for predicting DOS for TKA and THA [23,24]. Both models were trained on procedures from 2014 to 2017, hyperparameters tuned with Ray on procedures from 2018, and evaluated on procedures from 2019 [23,24,30,31]. These models were generated using the Niagara supercomputer at the SciNet HPC Consortium [32]. Predictions of DOS from the test subsets of data were used in the optimization model. Refer to the study by Abbas et al [23,24] for further details regarding the DOS prediction, model development, hyperparameter tuning, and feature importance.

### Schedule Optimization Formulations

#### Assumptions

The generated scheduling model considered elective case scheduling, in which surgeries are planned for in advance. The constraints and available resources for the model were generated using real-world constraints from the authors’ institution, the highest-volume elective arthroplasty hospital in Canada. This included cleaning time (30 min), number of operating rooms (n=5), number of surgeons (n=11), and days a surgeon was unavailable in a week. The planning horizon of 1 week (5 workdays) was also based on these constraints. The penalty for an individual operating room running overtime (after 5 pm), λ, was chosen to be double the value of daytime operating room underutilization. This was due to the approximate additional costs associated with operating room staff working after hours. The following assumptions regarding scheduling were made: surgeons are available to operate any day anytime except for 0‐2 randomly selected days per week per surgeon, and once a surgery is assigned to a surgeon, they must perform it (ie, no sharing patients). These assumptions were made as the study did not have access to historical data on surgeon availability from the authors’ institution. There were no constraints placed on the schedule based on staffing or patient beds in the recovery unit or ward.

#### Optimization Formulations

In total, 3 scheduling optimization problems were formulated. All formulations were based on an integer linear programming framework that has been used for many different scheduling problems, including but not limited to surgery scheduling [27,33]. The first, “Any,” was adapted from Marques et al [27] with notable modifications including the addition of an overtime penalty and only considering 1 surgery specialty. “Any” allows any surgery to be scheduled at any time of day in any room subject to the constraints that no surgeries in a room overlap, and that no surgeon is operating in 2 rooms simultaneously. The second formulation, “Split,” is the same as “Any” but enforces a maximum of 2 surgeons per operating room on a given day and a maximum of 1 room per surgeon per day. This was chosen as it is common when there are rooms that may be split between 2 surgeons if 1 surgeon cannot fill the room for that particular day. Finally, the third formulation is akin to a max-sum multiple subset sum problem and is thus referred to as “MSSP.” It enforces 1 surgeon per operating room on a given day, simplified into multiple optimization problems for each surgeon, following a fair distribution of rooms among surgeons. This is the most common configuration used on a clinical basis, where one surgeon has one operative room dedicated to their cases per day. “Any” is the most flexible optimization formulation, ie, the least constrained of the 3. “Split” and ”Any” impose additional constraints on feasible schedules and reflect realistic logistical restrictions that a hospital may want to impose, eg, having a surgeon use the same room on any given day. Details of these formulations are found in Multimedia Appendix 1.

#### Simulated Schedule Generation

Using the ACS NSQIP data, simulated schedules were generated using each optimization formulation, with schedule parameters as follows. Schedule granularity of 10- and 15-minute block times was considered. These sizes were chosen to ensure interpretable schedule generation. Surgery completion times were rounded up to the nearest block. The effect of surgeon waitlist size on schedule accuracy was also evaluated. Waitlist sizes representing 2 weeks (n=250), 4 weeks (n=500), 8 weeks (n=1000), and 12 weeks (n=1500) were considered. The cases within the waitlist were all given the same priority to be booked (no rank by time), unless randomly considered to be of high priority.

Three different versions of each model with the above schedule parameters were created. The first model used the ML-predicted DOS values for THA and TKA; this is the novel 2-stage approach of predicting then optimizing that we are proposing in this work. The second model used the mean DOS for each different type of surgery (THA and TKA) in order to obtain a schedule that mimics the current operating room schedule. Finally, a hindsight model was created that used the true DOS values to provide an upper bound on the best possible schedule that could be generated with perfect information (100% accurate to the minute). Each of these 3 simulations was performed 104 times (to represent 2 y) using a random sample of surgeries from the testing set.

#### Scheduling Comparisons

To compare the 3 schedule optimization formulations (Any, Split, and MSSP), the results of the 2-stage predict-then-optimize simulation results across all schedule parameter combinations were assessed. To evaluate the efficacy of the 2-stage schedule generation technique, it was compared to the results of scheduling by 2 other techniques: the mean DOS for each surgery (the current gold standard at most institutions) and the hindsight schedule. Each of these 3 schedules was constructed for each week of the simulation, and metrics consisting of overtime, underutilization, and the objective function value were calculated for each schedule. Student’s t-test was used to compare the effect between scheduling formulations. Overtime and underutilization for 2-stage and mean DOS schedules over the simulated weeks were compared using the paired Wilcoxon signed-rank test, as the same surgical cases were randomly selected for each generated schedule. The effect of schedule granularity and considered waitlist size was evaluated using unpaired Student’s *t*-test and analysis of variance as these were grouped over multiple selections of random cases. *P* values of <.05 were considered statistically significant.

## Results

### Prediction Model Accuracy

Demographic details of patients included are found in Multimedia Appendix 2. A summary of the DOS prediction models is found in Multimedia Appendix 3. The TKA prediction model obtained a training accuracy (with a 30 min buffer) of 76.9% and a training mean squared error (MSE) of 0.904. The validation accuracy was 77.7% with an MSE of 0.904. The test accuracy was 78.1% with an MSE of 0.898. The THA prediction model obtained a training accuracy of 74.0% and a training MSE of 0.888. The validation accuracy was 75.0% with an MSE of 0.910, and the test accuracy was 75.4% with an MSE of 0.916.

### Schedule Optimization Formulation Comparison

**Figure 2.**
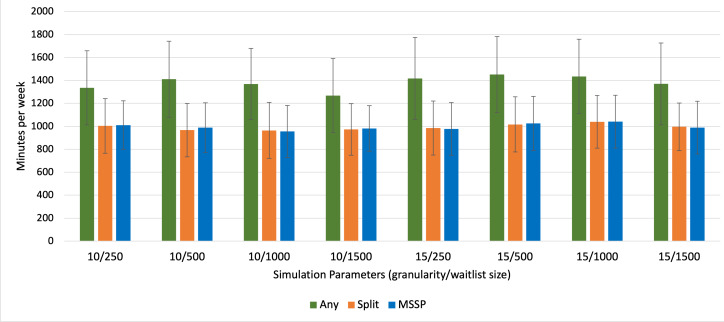
Mean overtime for each schedule optimization formulation across all schedule parameter combinations. MSSP: multiple subset sum problem.

Overall, the Any scheduling optimization formulation exhibited the poorest performance across all different schedule parameter combinations (Table 1). There was no significant difference in overtime across all schedule parameters between the Split and MSSP formulations. For 2 combinations of schedule parameters (10 min granularity, 1500 waitlist size and 15 min granularity, 1500 waitlist size), there was significantly less operating room underutilization with the MSSP formulation; however, this was only 10.9 minutes and 15.4 minutes, respectively, on average over an entire week (Table 1). In contrast, for 15-minute granularity and a 250-waitlist size, there was significantly more operating room underutilization with MSSP compared to the Split formulation, 42.3 minutes over an entire week (Table 1). Figures2  3 display this comparison for overtime and underutilization across all 3 optimization problems, respectively.

**Table 1.** Simulated schedule results for 2-stage (predict-then-optimize) for each schedule optimization formulation. The mean number of cases/week is not significant between any schedule optimization formulations.

Schedule parameters	Overtime min/week, mean (SD)	*P* value		Underutilization min/week, mean (SD)	*P* value		Mean cases/week (SD)
Granularity=10 minutes, waitlist=250 patients (2 wk)							
Any	1335.7 (322.1)	Reference	<.001	669.1 (189.1)	Reference	<.001	125.0 (0.2)
Split	1003.8 (238.1)	<.001	Reference	285.2 (117.4)	<.001	Reference	125.0 (0.2)
MSSP^a^	1010.9 (211.3)	<.001	.82	309.7 (128.7)	<.001	.15	124.2 (0.9)
Granularity=10 minutes, waitlist=500 patients (4 wk)							
Any	1410.4 (331.2)	Reference	<.001	694.4 (157.0)	Reference	<.001	125.0 (0.2)
Split	966.7 (232.9)	<.001	Reference	300.8 (113.9)	<.001	Reference	125.0 (0.1)
MSSP	988.6 (216.3)	<.001	.49	298.8 (119.8)	<.001	.91	124.9 (0.2)
Granularity=10 minutes, waitlist=1000 patients (8 wk)							
Any	1368.8 (310.0)	Reference	<.001	689.6 (183.7)	Reference	<.001	124.9 (0.3)
Split	964.0 (243.9)	<.001	Reference	301.6 (119.9)	<0.001	Reference	125.0 (0.2)
MSSP	954.9 (225.7)	<.001	.78	306.3 (112.8)	<0.001	.78	124.7 (0.6)
Granularity=10 minutes, waitlist=1500 patients (12 wk)							
Any	1267.9 (321.9)	Reference	<.001	657.7 (165.2)	Reference	<.001	124.8 (0.4)
Split	972.2 (225.2)	<.001	Reference	325.9 (114.8)	<.001	Reference	124.9 (0.3)
MSSP	981.3 (199.0)	<.001	.34	315.0 (109.9)	<.001	<.001	124.3 (0.7)
Granularity=15 minutes, waitlist=250 patients (2 wk)							
Any	1416.2 (357.7)	Reference	<.001	702.3 (185.5)	Reference	<.001	123.4 (1.8)
Split	985.1 (235.6)	<.001	Reference	307.6 (119.9)	<.001	Reference	123.1 (1.9)
MSSP	977.9 (229.7)	<.001	.82	349.9 (144.4)	<.001	.02	121.6 (1.5)
Granularity=15 minutes, waitlist=500 patients (4 wk)							
Any	1450.8 (331.3)	Reference	<.001	674.4 (167.6)	Reference	<.001	124.8 (0.4)
Split	1016.3 (240.1)	<.001	Reference	273.9 (104.0)	<.001	Reference	124.9 (0.3)
MSSP	1025.6 (235.9)	<.001	.78	263.7 (111.3)	<.001	.50	124.6 (0.7)
Granularity=15 minutes, waitlist=1000 patients (8 wk)							
Any	1434.7 (324.7)	Reference	<.001	669.7 (160.8)	Reference	<.001	124.6 (0.7)
Split	1039.3 (229.5)	<.001	Reference	242.0 (99.3)	<.001	Reference	124.9 (0.4)
MSSP	1040.8 (230.6)	<.001	.96	260.5 (100.1)	<.001	.19	124.8 (0.5)
Granularity=15 minutes, waitlist=1500 patients (12 wk)							
Any	1369.9 (356.1)	Reference	<.001	641.3 (153.5)	Reference	<.001	123.8 (1.2)
Split	996.2 (207.1)	<.001	Reference	294.1 (108.3)	<.001	Reference	124.5 (0.7)
MSSP	988.6 (229.9)	<.001	.59	278.7 (113.1)	<.001	.001	124.5 (0.7)

a MSSP: multiple subset sum problem.

**Figure 3.**
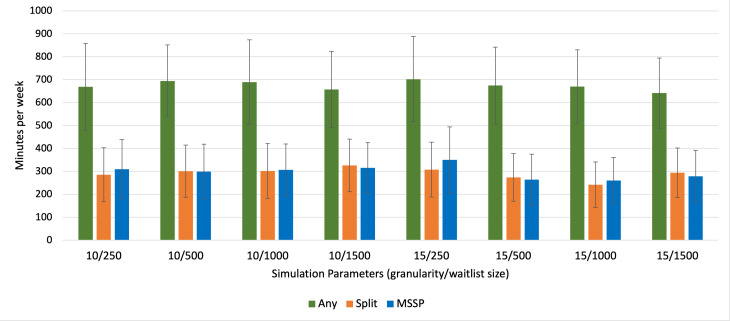
Mean underutilization for each schedule optimization formulation across all schedule parameter combinations. MSSP: multiple subset sum problem.

### Simulated Schedule Comparison

As the MSSP scheduling formulation performed best across all schedule parameters, it was used in all further analyses. The 2-stage predict-then-optimize approach performed better than using mean DOS for over 80% of weekly schedules in terms of objective optimization problem value across all schedule parameter combinations. This difference was more consistent across the schedules generated using 15-minute schedule granularity size, where 2-stage was superior to mean in over 90% of simulated schedules (Table 2). There was less overtime across all schedule parameters when using the 2-stage approach (*P*<.001), equating to an average decrease in overtime of 300-500 minutes per week at the simulated hospital (or 12-20 min per operating room per day). However, there was more operating room underutilization with the 2-stage approach across all schedule parameters (Table 3; *P*<.001). As expected, the hindsight schedule was nearly perfect for all generated schedules and was significantly better than the 2-stage or mean approach with respect to objective value, overtime, and underutilization (Table 3). Despite a statistically significant difference, there was no clinically realizable difference in the number of cases performed between the 2-stage and mean groups; however, the hindsight formulation scheduled less cases than both the mean and the 2-stage approach (mean of approximately 12 fewer cases per wk). Details regarding the Any and Split scheduling formulation results are found in MultimediaAppendices 4  5, respectively.

The changes to schedule granularity and considered waitlist size did not influence the amount of overtime. However, there was significantly less operating room underutilization with the 15-minute granularity schedules (*P*=.02) and with waitlist sizes greater than 500, or 1 month considered (*P*<.001) (Table 4).

**Table 2.** Percentage of simulations in which the 2-stage performed better than mean for all schedule parameters using the MSSP schedule optimization formulation.

Granularity	250 cases	500 cases	1000 cases	1500 cases
10 minutes	86.5	90.4	80.1	81.7
15 minutes	95.2	91.3	97.1	93.3

**Table 3.** Comparing 2-stage mean and hindsight durations of surgery using the multiple subset sum problem (MSSP) schedule optimization formulation.

Method	Overtime (min/week), mean (SD)	*P* value	Underutilization (min/week), mean (SD)	*P* value	Mean cases/week (SD)	*P* value
Granularity=10 minutes, waitlist=250 patients (2 wk)						
Two-stage	1010.9 (211.3)	Reference	309.7 (128.7)	Reference	124.2 (0.9)	Reference
Mean	1310.9 (296.9)	<.001	218.7 (99.1)	<.001	124.9 (0.5)	<.001
Hindsight	0.4 (3.1)	<.001	13.8 (52.8)	<.001	112.5 (2.8)	<.001
Granularity=10 minutes, waitlist=500 patients (4 wk)						
Two-stage	988.6 (216.3)	Reference	298.8 (119.8)	Reference	124.9 (0.2)	Reference
Mean	1305.7 (242.2)	<.001	220.8 (102.0)	<.001	125.0 (0)	.01
Hindsight	0 (0)	<.001	0 (0)	<.001	112.8 (2.7)	<.001
Granularity=10 minutes, waitlist=1000 patients (8 wk)						
Two-stage	954.9 (225.7)	Reference	306.3 (112.8)	Reference	124.7 (0.6)	Reference
Mean	1276.3 (310.1)	<.001	220.4 (101.5)	<.001	125.0 (0)	<.001
Hindsight	0 (0)	<.001	0 (0)	<.001	120.3 (3.4)	<.001
Granularity=10 minutes, waitlist=1500 patients (12 wk)						
Two-stage	981.3 (199.0)	Reference	315.0 (109.9)	Reference	124.3 (0.7)	Reference
Mean	1267.7 (224.0)	<.001	223.4 (95.1)	<.001	125.0 (0)	.24
Hindsight	0 (0)	<.001	0.0 (0)	<.001	125.4 (3.9)	.006
Granularity=15 minutes, waitlist=250 patients (2 wk)						
Two-stage	977.9 (229.7)	Reference	349.9 (144.4)	Reference	121.6 (1.5)	Reference
Mean	1485.0 (274.0)	<.001	158.1 (107.4)	<.001	124.9 (0.5)	<.001
Hindsight	0.4 (3.3)	<.001	15.7 (66.2)	<.001	113.8 (2.5)	<.001
Granularity=15 minutes, waitlist=500 patients (4 wk)						
Two-stage	1025.6 (235.9)	Reference	263.7 (111.3)	Reference	124.6 (0.7)	Reference
Mean	1526.7 (275.6)	<.001	149.3 (81.9)	<.001	125.0 (0)	<.001
Hindsight	0 (0)	<.001	0 (0)	<.001	116.1 (2.5)	<.001
Granularity=15 minutes, waitlist=1000 patients (8 wk)						
Two-stage	1040.8 (239.8)	Reference	260.5 (100.1)	Reference	124.8 (0.5)	Reference
Mean	1562.2 (262.0)	<.001	140.6 (90.7)	<.001	125.0 (0)	<.001
Hindsight	0 (0)	<.001	0 (0)	<.001	119.0 (2.1)	<.001
Granularity=15 minutes, waitlist=1500 patients (12 wk)						
Two-stage	988.6 (229.9)	Reference	278.7 (113.1)	Reference	124.5 (0.7)	Reference
Mean	1540.0 (267.0)	<.001	164.6 (79.8)	<.001	125.0 (0)	<.001
Hindsight	0 (0)	<.001	0 (0)	<.001	118.8 (2.4)	<.001

**Table 4.** Comparing the impact of schedule parameters for the 2-stage multiple subset sum problem (MSSP).

Schedule parameters	Overtime, min/week, mean (SD)	*P* value	Underutilization, min/week, mean (SD)	*P* value
Granularity		.12		.02
10 min	983.9 (213.9)		307.5 (117.8)	
15 min	1008.2 (231.5)		288.2 (117.2)	
Waitlist size		.79		<.001
250 (2 weeks)	994.4 (221.9)		329.8 (138.5)	
500 (4 weeks)	1007.1 (227.6)		281.25 (117.2)	
1000 (8 weeks)	997.8 (232.8)		283.4 (109.4)	
1500 (12 weeks)	984.9 (215.5)		296.8 (113.3)	

## Discussion

This paper compared 3 different scheduling optimization problems and evaluated a novel approach to surgical scheduling for TKAs and THAs using a combined 2-stage ML DOS prediction and optimization. There was no significant difference in operating room underutilization or overtime between the MSSP (one surgeon designated to one operating room per day) or Split (maximum of 2 surgeons designated for 1 operating room per day) optimization formulations. However, both performed significantly better than the Any (no limit on surgeons per operating room per day) formulation.

We believe this is due to the limitations of the predictions: underestimating the DOS of 1 case c being performed by surgeon h in room r can have cascading effects on another room r^′^ in which the same surgeon h is due to perform another surgery c^′^ at a later time. This causes additional overtime penalties for “Any,” something that the more restrictive “Split” and “MSSP” do not encounter. This is why, despite optimal solutions to “Any” being theoretically better than those of “Split,” they performed worse when simulated with the actual DOS.

Overall, the combined 2-stage approach significantly outperformed the current standard for scheduling cases, which is a case-specific mean surgery duration. This performance improvement was maintained across all schedule parameter combinations, including different schedule block granularity and different patient waitlist sizes considered. Despite this improvement, the 2-stage approach performed considerably worse than the hindsight schedule, highlighting the limitations of the current predictions that are based solely on preoperative patient data. Interestingly, there was no impact on overtime by varying schedule granularity or waitlist size; however, both of these impacted the amount of underutilization. The smaller waitlist size of 2 weeks of considered cases had a greater amount of underutilization, which was likely due to surgeons not having enough cases to fill their operating room time. Once a threshold was met at a 4-week pool of cases, there was no difference between groups. Also notable was the fact that when only considering a 2-week pool of cases, the Split optimization formulation outperformed MSSP, likely due to some surgeons not having enough cases to fill their time. Therefore, as the MSSP formulation is most practically implementable, it must be ensured that either the considered case pool is large enough or surgeons are allocated time when they have enough cases to fill an entire operating room day to avoid underutilization.

Errors in procedure length estimation by clinicians occur in approximately 75% of cases, with 32%‐50% of daily operating room schedules being underbooked and 37%‐42% overbooked [34,35]. This is compounded by the fact that less than 50% of operating time is spent doing surgery [34]. Booking based on a historical mean is more accurate than when estimated by the surgical team, though less accurate than traditional ML approaches [10,36-38]. Previous approaches using computing to improve surgical scheduling have included schedule optimization or ML to predict DOS in isolation [13,21,27,37,39,40]. Other approaches to scheduling operating room utilization include the use of surgeon-specific mean DOS or a surgeon case-specific rolling average time. To our knowledge, these have not been compared to an ML prediction-based approach. Due to the lack of surgeon-specific details included in the ACS NSQIP dataset, we could not assess the efficacy of these approaches in the present study.

The implementation of this predict-then-optimize scheduling approach would face several challenges in the real world. The MSSP optimization model is in line with current surgical scheduling practices at most hospitals. This formulation optimizes a specific surgeon’s waiting list, increasing their ability to accurately plan their day while ensuring a fair distribution of time (by operating room days) for each surgeon. However, attempting to implement the other optimization formulations (Split and Any) may be faced with resistance by end-users. Particularly, using the Any formulation, surgeons may have cases at the beginning and end of the day spread out across more days in a week. ML-predicted DOS has been trialed previously in operating room planning by one group that found a reduction in wait time between cases [41]. However, they generated the predicted DOS and evaluated the implications of that information over a single day, not considering other cases from the waitlist or optimizing the schedule based on the predicted DOS.

In addition to improving operating room utilization at the systems level, the present study has implications for surgeon-level daily planning. Accurate patient-specific DOS prediction and scheduling allow for more effective personal scheduling, including preoperative preparation, intraoperative workflow, and postoperative responsibilities. Accurate DOS predictions can assist surgeons in anticipating the need for ancillary support (eg, anesthesia, nursing, imaging) and have the potential to help decrease fatigue associated with unplanned overruns. When integrated into scheduling systems that allocate block time based on surgeon-specific waitlists (as in the MSSP formulation), this can enhance both surgeon efficiency and case throughput, aligning institutional resource allocation with the surgeon’s realistic operative capacity.

The generated models and optimization formulations have the ability to transform how elective operating room scheduling is performed. By developing models specific to each operation, this increases the accuracy of each model. Most previous research evaluating the effect of ML for DOS prediction has grouped multiple different procedures [21,37]. Using such models, the procedure performed would generally be the most important feature, diluting the effect of other important patient factors without using appropriate ML techniques. The potential for cost savings for hospitals, related to reduction in overtime costs and valuable underused operating room time, is high, but the main limitation lies in the accuracy of the DOS prediction. The predictive models for TKA and THA included 33 individual patient features. Improving the model with the use of operational factors from a specific institution would likely have a corresponding effect on the schedule results but would reduce the generalizability of the approach. Further improvements may also be made by directly integrating the downstream scheduling optimization problem into training the predictive ML model [42].

This work presents some limitations. First, the DOS prediction was restricted to preoperative patient factors, based on the availability of data elements in the ACS NSQIP database, limiting the prediction accuracy. This was evidenced by the large difference in schedule performance between the 2-stage method and the perfect, hindsight, schedule. Nevertheless, this approach to predicting DOS still yielded improved schedules as compared to using a surgery-specific mean time estimate. Second, the goal of our optimization problem was to maximize the utilization of the operating room; however, this may not be directly in line with the goals of all hospitals, as some institutions may have other priorities, such as maximizing the number of cases completed. This project only developed predictive models for primary TKA and THA procedures, which may have artificially lowered the potential effect size of using this approach, as these are relatively routine procedures with lower DOS variability. By generating more surgery-specific predictive models within orthopedic surgery or other specialties, the potential for this approach may be even larger. However, these results are more directly applicable to high-volume arthroplasty surgical centers. Additionally, this scheduling approach was a simplified proof-of-concept model that may not be applicable to more complex, real-world scheduling scenarios. For example, it did not consider downstream constraints, such as the number of recovery beds, ward beds, or available staff. This was not a concern at our local institution due to its relative efficiency as a high-volume arthroplasty center. However, this would need to be expanded upon in future work if this is to be practically implemented in less specialized centers. Finally, the Split and Any optimization problems were computationally intensive when considering a waiting list size of 2‐3 months of patients. This may be a consideration depending on institutional computational resources, which is worth noting when implementing a similar solution.

In conclusion, using ML patient-specific DOS predictions coupled with optimization was superior to elective scheduling based on a mean DOS metric over 3 different optimization problems with varying constraints and combinations of waitlist size and granularity. This generalizable approach suggests that improvements in hospital resource utilization are possible with the application of new computational methods, but the inclusion of institution-specific operational data may be considered to further improve predictions and scheduling. This has significant potential implications for health care systems struggling with pressures of rising costs and growing operative waitlists.

## Supplementary material

10.2196/70857 Multimedia Appendix 1 Optimization formulations.

10.2196/70857 Multimedia Appendix 2 Select preoperative continuous, categorical, and ordinal feature distributions of total knee and hip arthroplasty patients.

10.2196/70857 Multimedia Appendix 3 Results of the training, validation, and testing set for the duration of surgery (DOS) predictions for total knee and hip arthroplasty (TKA and THA) models, respectively. Accuracies are presented in percentages.

10.2196/70857 Multimedia Appendix 4 Comparing 2-stage, mean, and hindsight duration of surgery using the “Any” schedule optimization formulation.

10.2196/70857 Multimedia Appendix 5 Comparing 2-stage, mean, and hindsight duration of surgery using the “Split” schedule optimization formulation.

## Abbreviations

ACS NSQIP: American College of Surgeons National Surgical Quality Improvement
DOS: duration of surgery
ML: machine learning
MSSP: multiple subset sum problem
THA: total hip arthroplasty
TKA: total knee arthroplasty
