# Supplementary material for: Using Machine Learning to Predict-Then-Optimize Elective Orthopedic Surgery Scheduling to Improve Operating Room Utilization: Retrospective Study
Source: JMIR Med Inform. 2025 Sep 10;13:e70857. doi: 10.2196/70857 (PMC12422739; doi:10.2196/70857)
Supplement: Multimedia Appendix 1 [file medinform-v13-e70857-s001.docx]

### Multimedia Appendix 1: Optimization Formulations.

### Notation

We use C for the set of all surgeries under consideration, R for the set of all operating rooms, and D for the set of all days in the planning horizon. Time is discretized into slots with granularity g (e.g., 10 minutes or 15 minutes). T_c_ is the set of all time slots in a day and T_o_ is the subset of T_c_ consisting of overtime slots (e.g., after 5pm), for which a penalty will be applied. p_c_ is the duration of surgery for surgery c divided by granularity g. γ is the time needed for cleaning the rooms between operations divided by granularity g. λ is the weight for the penalty of going overtime. C_2_ is the set of surgeries that have high priority, i.e., that must be scheduled mandatorily in the planning horizon. The subscript of 2 in C_2_ is used to be consistent with past work done to define priority groups^24^. H is the set of all surgeons, and h_c_ is the surgeon that is assigned to surgery c. Binary indicators i_hd_ are used to denote whether surgeon h is available on day d.

### “Any” formulation


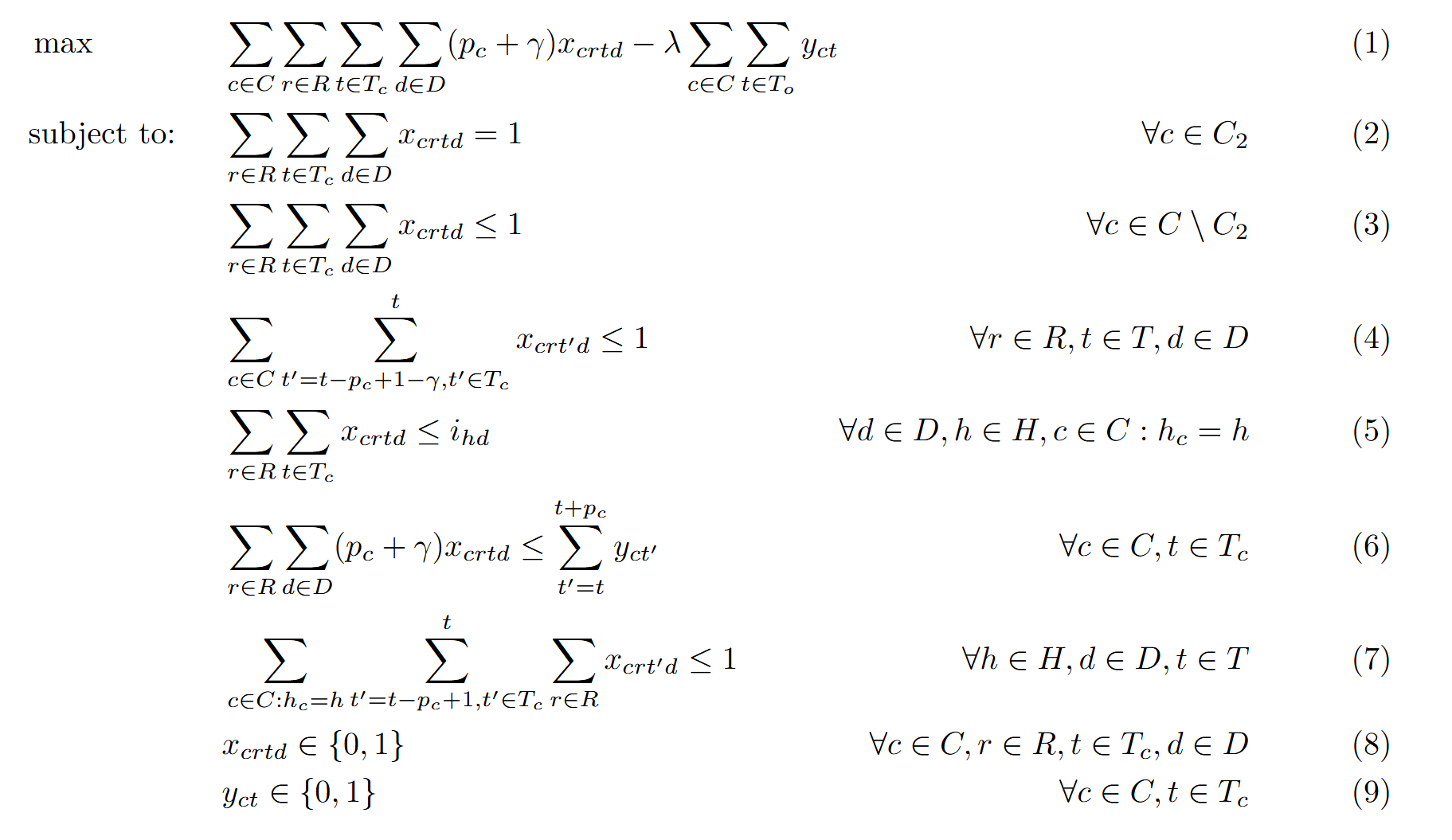


This is a scheduling problem with both soft and hard time constraints. The soft constraint allows surgeries to be scheduled over OR closing time, incurring an overtime penalty. However, there is also a hard constraint of 1 hour after closing that is reflected in T_c_. The objective (1) is to maximize the total OR utilization time (first term) while penalizing any overtime using a penalty term, λ (second term). The decision variables defined in Constraints (8, 9) are x_crtd_ and y_ct_, all of which are binary, where x_crtd_ is 1 if surgical case c is assigned to room r and performed starting at time slot t on day d, and y_ct_ is 1 if surgery c is performed in time slot t. Constraint (2) ensures that patients with high priority are scheduled within the planning horizon. Constraint (3) enforces that a surgery can only be done once. Constraint (4) ensures that there is no overlap between surgeries in a room, including a cleaning time of γ. Constraint (5) ensures that a surgery cannot be scheduled if the surgeon is unavailable on that day. Constraint (6) defines the relation between x and y. Constraint (7) ensures that there is no overlap between surgeries performed by the same surgeon across different rooms.

### “Split” formulation


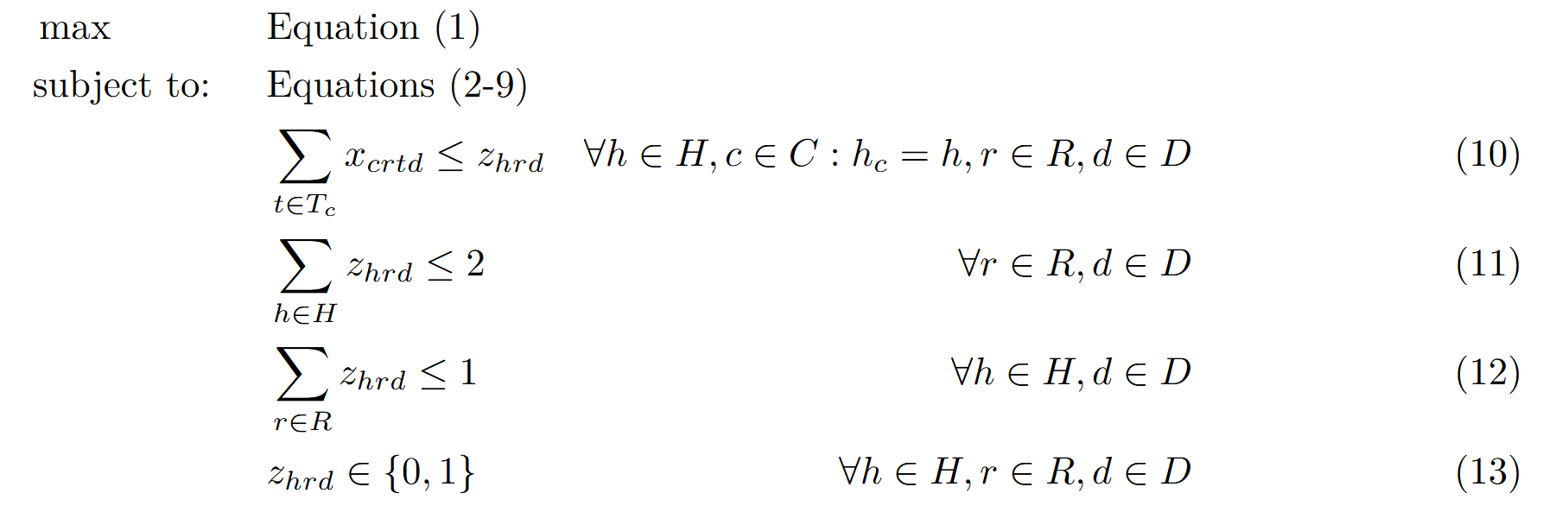


“Split” is almost identical to the Any formulation, with a few modifications. A new binary variable, z_hrd_, is introduced in Constraint (13) to define whether a surgeon h is performing any surgeries in room r on day d. Constraint (10) defines the relation between x and z. Constraint (11) ensures that there are at most 2 surgeons performing surgery in any one operating room, while Constraint (12) ensures that a surgeon only performs surgery in one room on a given day.

### Max-Sum Multiple Subset Sum Problem (“MSSP”) formulation


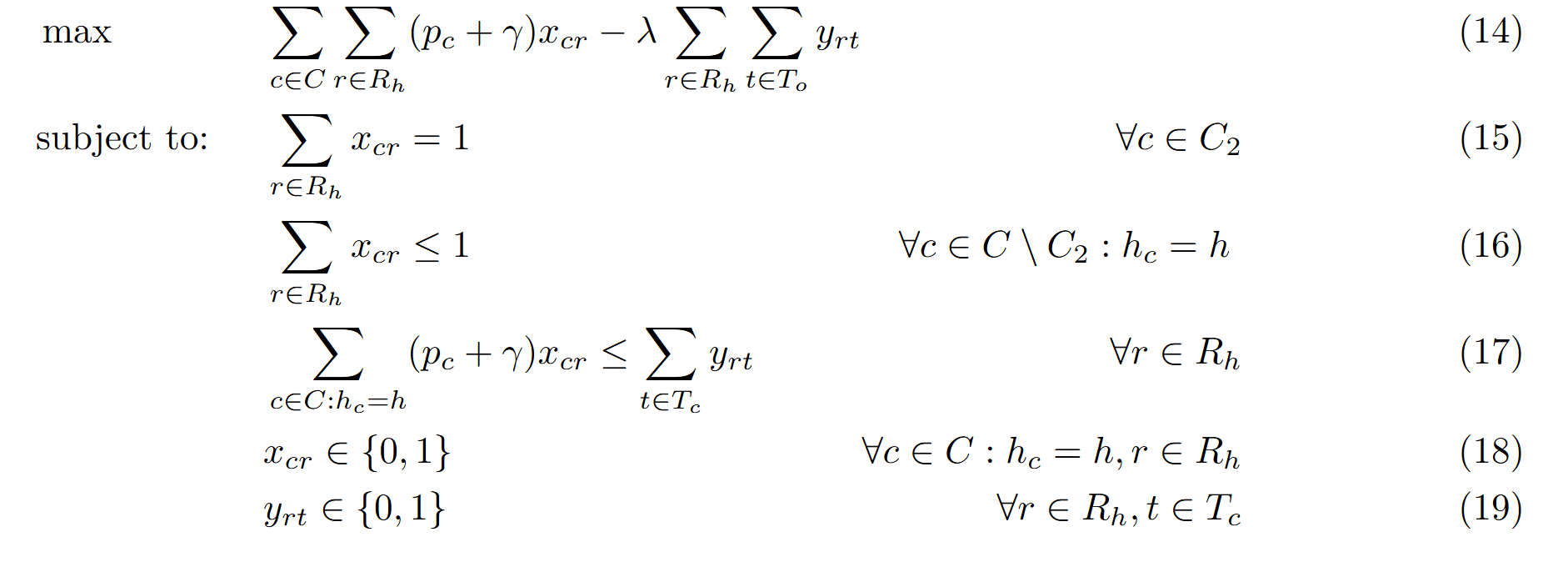


The MSSP optimization problem was solved for each surgeon’s waitlist, and then the results of each optimization problem were combined into one schedule. The solution is independent of the order in which each subpart of the MSSP problem was solved, as the operating room assignments are done prior to the solve. Operating rooms were assigned to each surgeon by giving each surgeon an equal number of rooms, with any surplus rooms assigned to the surgeons with the most availability.

The two sets of binary decision variables are x_cr_, which is set to 1 if a surgery c is performed in room r, and y_rt_, which is set to 1 if a surgery is being performed in room r at time slot t. In this formulation, R_h_ refers to the rooms that are assigned in advance to each surgeon; each room is used on a different day. The objective (14) is similar to the one from “Any” and “Split”. Constraint (15) ensures that the priority cases are done in the planning horizon, and Constraint (16) ensures that a surgery is performed at most once. Constraint (17) defines the relationship between x and y.
