# Supplementary material for: Using Machine Learning to Predict-Then-Optimize Elective Orthopedic Surgery Scheduling to Improve Operating Room Utilization: Retrospective Study
Source: JMIR Med Inform. 2025 Sep 10;13:e70857. doi: 10.2196/70857 (PMC12422739; doi:10.2196/70857)
Supplement: Multimedia Appendix 2 [file medinform-v13-e70857-s002.docx]

### Multimedia Appendix 2: Supplementary Table 1

**Supplementary Table 1.** Select preoperative continuous, categorical, and ordinal feature distributions of total knee and hip arthroplasty patients.

| **Variable** | **Total knee arthroplasty (N = 302,490)**  **N (%)** | **Total hip arthroplasty**  **(N = 196,942)  N (%)** | **P-value** |
| --- | --- | --- | --- |
| Age (years) — mean (SD) | 67.0 (9.3) | 65.3 (11.3) | .011 |
| Weight (kg) — mean (SD) | 91.8 (20.9) | 85.9 (20.7) | <.001 |
| Height (cm) — mean (SD) | 166.5 (10.7) | 168.1 (10.4) | .001 |
| Body mass index (kg/m^2) — mean (SD) | 33.0 (6.7) | 30.3 (6.2) | <.001 |
| Female sex | 186,318 (61.6) | 107,893 (54.8) | <.001 |
| Current smoker | 24,272 (8.0) | 24,548 (12.5) | .003 |
| On dialysis | 467 (0.2) | 446 (0.2) | .023 |
| Disseminated cancer | 345 (0.1) | 654 (0.3) | .002 |
| Chronic obstructive pulmonary disease | 10,231 (3.4) | 7,540 (3.8) | <.001 |
| Congestive heart failure | 972 (0.3) | 686 (0.3) | .003 |
| Hypertension | 194,900 (64.5) | 108,468 (55.1) | <.001 |
| Acute renal failure | 62 (0.02) | 104 (0.05) | <.001 |
| Steroid use for chronic condition | 10,680 (3.5) | 7,315 (3.7) | <.001 |
| Major weight loss^a^ | 280 (0.1) | 397 (0.2) | <.001 |
| Blood transfusion up to 72 hours preop | 67 (0.02) | 247 (0.1) | <.001 |
| Bleeding disorder | 5,757 (1.9) | 3,959 (2.0) | <.001 |
| Race |  |  | .002 |
| White | 218,970 (72.3) | 144,037 (73.1) |  |
| Unknown | 50,562 (16.7) | 33,468 (17.0) |  |
| Black or African American | 23,789 (7.9) | 15,330 (7.8) |  |
| Asian | 6,269 (2.1) | 2,830 (1.4) |  |
| Native American | 2,710 (0.9) | 1,322 (0.7) |  |
| Primary Anesthesia Technique |  |  | <.001 |
| General | 127,482 (42.7) | 92,408 (46.9) |  |
| Spinal | 121,181 (40.1) | 72,296 (36.7) |  |
| MAC/IV Sedation | 46,484 (15.4) | 28,338 (14.4) |  |
| Regional | 5,261 (1.7) | 2,940 (1.5) |  |
| Epidural | 1,892 (0.6) | 1,005 (0.5) |  |
| Diabetes |  |  | <.001 |
| No | 247,010 (81.7) | 173,011 (87.8) |  |
| Managed without insulin | 42,185 (14.0) | 18,339 (9.3) |  |
| Managed with insulin | 13,105 (4.3) | 5,637 (2.9) |  |
| Dyspnea |  |  | .003 |
| No | 285,808 (94.5) | 188,401 (95.6) |  |
| Moderate exertion | 15,982 (5.3) | 8,246 (4.2) |  |
| At rest | 510 (0.2) | 340 (0.2) |  |
| Functional status |  |  | .004 |
| Independent | 299,469 (99.1) | 193,817 (98.4) |  |
| Partially dependent | 2,757 (0.9) | 3,040 (1.5) |  |
| Totally dependent | 74 (0.1) | 130 (0.1) |  |

^a^Defined as >10% weight loss within 6 months of surgical procedure.
