# Supplementary material for: Using Machine Learning to Predict-Then-Optimize Elective Orthopedic Surgery Scheduling to Improve Operating Room Utilization: Retrospective Study
Source: JMIR Med Inform. 2025 Sep 10;13:e70857. doi: 10.2196/70857 (PMC12422739; doi:10.2196/70857)
Supplement: Multimedia Appendix 3 [file medinform-v13-e70857-s003.docx]

### Multimedia Appendix 3: Supplementary Table 2

**Supplementary Table 2.** Results of the training, validation, and testing set for the duration of surgery (DOS) predictions for total knee and hip arthroplasty (TKA and THA) models, respectively. Accuracies represented in percentages.

| **Model** | **MSE** | **Accuracy**  **(15 min)** | **Buffer accuracy**  **(30 min)** | **Buffer accuracy**  **(45 min)** | **Buffer accuracy**  **(60 min)** |
| --- | --- | --- | --- | --- | --- |
| Training (2014-2017) | | | | | |
| TKA | 0.904 | 44.7 | 76.9 | 91.8 | 95.9 |
| THA | 0.888 | 43.7 | 74.0 | 89.0 | 94.8 |
| Validation (2018) | | | | | |
| TKA | 0.904 | 45.1 | 77.7 | 91.9 | 96.0 |
| THA | 0.910 | 45.2 | 75.0 | 89.4 | 95.5 |
| Testing (2019) | | | | | |
| TKA | 0.898 | 46.6 | 78.1 | 91.5 | 96.2 |
| THA | 0.916 | 45.1 | 75.4 | 90.2 | 96.0 |
